# Estimation of Biological Oxygen Demand and Chemical Oxygen Demand for Combined Sewer Systems Using Synchronous Fluorescence Spectra

**DOI:** 10.3390/s100402460

**Published:** 2010-03-24

**Authors:** Jin Hur, Bo-Mi Lee, Tae-Hwan Lee, Dae-Hee Park

**Affiliations:** 1 Department of Earth and Environmental Sciences, Sejong University, 98 Gunja-dong, Gwangjin-gu, Seoul, 143-747, Korea; E-Mails: ch20610@hotmail.com (B.-M.L.); zdrc83@hanmail.net (T.-H.L.); 2 Locus Solution Co., Ltd., DMC High-Tech Center, Seoul, 121-270, Korea; E-Mail: danny@locuss.co.kr

**Keywords:** sewer system, fluorescence, prediction, BOD, COD

## Abstract

Real-time monitoring of water quality for sewer system is required for efficient sewer network design because it provides information on the precise loading of pollutant to wastewater treatment facilities and the impact of loading on receiving water. In this study, synchronous fluorescence spectra and its first derivatives were investigated using a number of wastewater samples collected in sewer systems in urban and non-urban areas, and the optimum fluorescence feature was explored for the estimation of biochemical oxygen demand (BOD) and chemical oxygen demand (COD) concentrations of sewer samples. The temporal variations in BOD and COD showed a regular pattern for urban areas whereas they were relatively irregular for non-urban areas. Irrespective of the sewer pipes and the types of the areas, two distinct peaks were identified from the synchronous fluorescence spectra, which correspond to protein-like fluorescence (PLF) and humic-like fluorescence (HLF), respectively. HLF in sewer samples appears to be associated with fluorescent whitening agents. Five fluorescence characteristics were selected from the synchronous spectra and the first-derivatives. Among the selected fluorescence indices, a peak in the PLF region (*i.e.*, Index I) showed the highest correlation coefficient with both BOD and COD. A multiple regression approach based on suspended solid (SS) and Index I used to compensate for the contribution of SS to BOD and COD revealed an improvement in the estimation capability, showing good correlation coefficients of 0.92 and 0.94 for BOD and COD, respectively.

## 1. Introduction

The water quality of combined sewer systems is affected by the lifestyle of the inhabitants as well as the temporal flow pattern (e.g., storm events) [1]. It may also be significantly changed by other factors such as the infiltration of groundwater, the leakage of pipes, the distance, and the hydraulic gradient [2]. In general, the construction and/or the replacement of sewer systems are planned and implemented after the information was collected on the sewage flow, the mass loading of pollutants, and condition of the pipes. Such a pre-evaluation step can help to avoid excessive loadings to other sewer lines and other undesirable environmental impact during the project.

Obtaining the high time resolution about water quality data for combined sewer overflows is also important for the proper evaluation of the influence of storm water flows on wastewater treatment plants as well as for the assessment of its impact on receiving waters. In addition, it provides the basis for the construction of mathematical modeling for the simulation of the two above-mentioned behaviors and the influences [3]. Variations in waste water quality are relatively large and abrupt changes may take place due to infiltration, leakage and storm events. Biochemical oxygen demand (BOD) and chemical oxygen demand (COD), indirect indicators of organic matters, are representative parameters for sewer water quality. However, it is very difficult to obtain continuous water quality data because of the scarcity of accessible space within the sewer systems and the necessity of separate laboratory experiments. Moreover, at least five days are required to acquire BOD data from the experiment and BOD itself may be biased by the presence of toxic substances that might cause the inhibition of the oxidizing bacteria.

Recently, optical techniques such as UV-visible spectroscopy and fluorescence measurements were suggested as fast and versatile monitoring tools for BOD and COD in water samples [4]. It is well known that UV absorbance at 250–300 nm is correlated with the concentrations of organic matters in sewage samples [5]. It is reported that PLF intensity is associated with fluorescent biodegradable organic compounds such as tryptophan and tyrosine [6–8]. The PLF of water samples exhibited a good relationship with BOD concentration and it showed the superiority in sensitivity and resolution over UV absorbance [7,9]. Fluorescence excitation-emission matrix (EEM) has been a preferred scanning method for monitoring and characterizing organic matters in water and wastewater samples until now. This is because it can capture all fluorophores contained in the samples [8,10].

In contrast to the fluorescence EEM that requires a number of scanning, synchronous fluorescence spectra can be obtained by only one scanning operation and most importantly, it still contains much information on the composition of organic matters. For example, Hur and Kong [11] reported a significant correlation between some selected fluorescence characteristics from synchronous fluorescence spectra and the BOD values for urban river samples affected by the effluent of a wastewater treatment plant.

The objectives of this study were: (1) to compare the temporal changes in BOD and COD concentrations and the synchronous fluorescence spectra of sewage samples from sewer pipes for urban versus non-urban areas; and (2) to suggest the optimum fluorescence indices for the estimation of BOD and COD based on the correlations between selected fluorescence features and the water quality data.

## 2. Experimental Section

### 2.1. Sample Collection and Preservation

Wastewater samples were collected from four different sewer pipes located in two separate urban areas (City A and B) and from nine sewer pipes in two non-urban districts. Grab sampling was conducted at manholes or accessible sewer pipe outlets on different dates from November 2008 to February 2009. For each sewer pipe, the sampling continued for at least 18 hours and the time interval remained 2–6 hours at low flow period. The populations of the cities A and B are approximately 140,000 and 200,000 respectively, whereas those of non-urban areas are less than 20,000. The samples were taken to the laboratory and stored in a refrigerator. BOD and COD experiments were conducted on the sampling dates and the other analyses were taken within 48 hours after the arrival of the samples.

### 2.2. Analytical Methods

BOD concentrations were determined following a standard protocol [12]. COD measurements were made using low- and high-range ampoules (HACH Chemical) with a spectrophotometer (HACH, DR5000). Synchronous fluorescence spectra of unfiltered samples were measured using a luminescence spectrometer with a 20 kW xenon arc lamp (Perkin-Elmer LS-50B). Excitation and emission slits were adjusted to 10 nm and 10 nm, respectively. The excitation wavelengths ranging from 250 to 600 nm were used with constant offsets (Δλ = 30 nm) [9,11]. When the UV absorbance of the samples at 254 nm was above 0.1, the corresponding samples were diluted prior to the fluorescence measurement to avoid the inner-filter correction and also to maintain the concentration within the range of measurable fluorescence intensity [8,9]. To limit second-order Raleigh scattering, a 290-nm cutoff filter was used for all samples. The fluorescence response to a blank solution (Milli-Q water) was subtracted from the spectra of each sample. The measured fluorescence intensities were then normalized to units of quinine sulfate equivalents (QSE) in μg/L using the fluorescence of a diluted series of quinine sulfate dehydrate in 0.05 M sulfuric acid at an excitation/emission of 350/450 nm [13] to reflect the stability of the instrument. Relative precisions of <2% were routinely obtained by repeating the fluorescence measurements of the samples.

To obtain additional information from the spectroscopy, first-order derivative fluorescence spectra (*i.e.*, dA(λ)/dλ) were calculated following the method of Hur *et al*. [14] using Origin 6.0 (Microcal Software, Inc.). Briefly, the process involved a stepwise interval smoothing of the original zero-order spectra using a selected order polynomial function followed by differentiation of that function to obtain the derivative spectra. For the present study, we selected a constrained second-order polynomial function smoothing using a 17-data point interval to calculate the first derivative [14].

## 3. Results and Discussion

### 3.1. Temporal Variations in BOD and COD Concentrations

**Figure 1.**
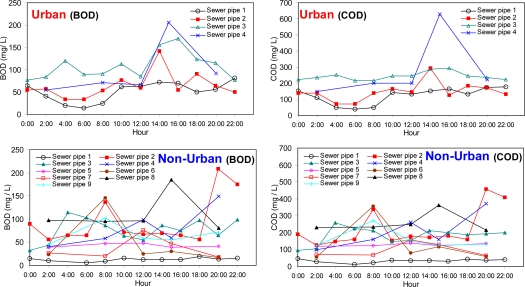
Temporal variations in BOD and COD concentrations of the sewer samples from sewer pipes for urban and non-urban areas.

In urban areas, BOD and COD concentrations exhibited a typical pattern of hourly variation with the highest peak at early afternoon, ranging from 13.6 mg/L to 206 mg/L and from 40 mg/L to 629 mg/L, respectively (Figure 1). In contrast, the patterns for non-urban areas were rather irregular with no common feature observed for the individual sewer pipe. The BOD and the COD concentrations ranged from 5.2 mg/L to 208 mg/L and from 13 mg/L to 456 mg/L, respectively. There was no significant difference in the range of BOD concentrations between the urban and the non-urban areas. For most sewer pipes of the non-urban areas, the highest concentrations of BOD and COD were observed at either early morning time or late nighttime (Figure 1). The observed difference in the temporal pattern appears to be caused by the dissimilarity in the lifestyles of the inhabitants in the urban areas and the non-urban areas. Alternatively, it may be explained by different level of the land cover. In general, urbanization decreases the permeability of land surface, leading to a decrease in infiltration and an increase in surface runoff [15]. More uncovered land in the non-urban areas is presumably related to the irregular temporal patterns in the water quality. Comparison of the range in the ratio of BOD to COD (*i.e.*, BOD/COD), which is interpreted as biodegradability for the non-urban versus the urban areas, showed that the sewage composition of the sewer pipes located in the non-urban areas might be more heterogeneous than in the urban areas. The BOD/COD ratios ranged from 0.26 to 0.77 for the non-urban areas whereas the urban areas exhibited much smaller range from 0.32 to 0.58.

### 3.2. Comparison of Synchronous Fluorescence Spectra

**Figure 2.**
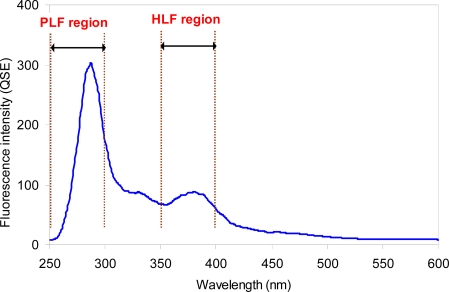
Typical synchronous fluorescence spectrum of the sewer sample from the sewer systems (Δ*λ* = 30 nm).

The typical synchronous fluorescence spectrum of collected sewer sample is shown in Figure 2. No significant difference in the shapes of the spectra was observed for the two types of areas (*i.e.*, urban and non-urban areas). Regardless of the degree of urbanization, two fluorescence peaks could be identified from the spectra. The first peak, related to the presence of fluorescent proteins and amino acids, was observed at the PLF region that is designated to the wavelength range between 250 nm and 300 nm. Another peak was found at a wavelength of ∼375 nm, which is designated to HLF for this study. The two observed fluorescence features have been often reported for other sewage samples [9,11,16,17]. The prominent peak in the PLF region is known to be a representative fluorescence characteristic of sewage, serving to discriminate clean river water [18]. The peak is reported to be closely correlated with microbial activities. Although HLF feature is typically attributed to the presence of humic acid, the HLF peak observed for this study appears to be more associated with fluorescent whitening agents present in sewage. This is because whitening agents are widely used as detergent additives, and fluorescence regions of humic acid and whitening agents overlap each other. In addition, the observable level of the peak is unusual even considering river samples rich in humic acids [9,17,19]. It is reported that high fluorescence intensity near the HLF region is observed from tissue mill effluent samples, which contain abundant whitening agents [20].

### 3.3. Temporal Variations in Protein-Like Fluorescence and Humic-Like Fluorescence Intensities

**Figure 3.**
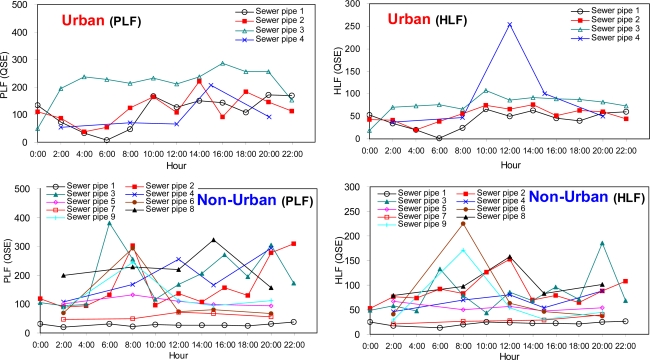
Temporal variations in protein-like fluorescence (PLF) and humic-like fluorescence (HLF) intensities (QSE unit) of the sewer samples from sewer pipes for urban and non-urban areas.

The temporal variations in PLF and HLF peak intensities were similar to those previously observed for BOD and COD concentrations (Figure 3). For unban areas, both PLF and HLF showed their highest intensities from 10:00 to 16:00 during the day. The unusual high peak of HLF observed from the sewer pipe 4 of the urban areas appears to be due to the rapid increase in the use of washing detergents at the short time period. Takahashi and Kawamura [19] used a HLF characteristic as a tracer of a whitening agent and they suggested a fluorescence monitoring method to estimate domestic wastewater ratio in river water. For non-urban areas, the temporal variations in the fluorescence characteristics were rather irregular and they showed different patterns for the individual sewer pipes (Figure 3). The dissimilarity in the temporal pattern of the selected fluorescence intensities was more pronounced for the non-urban areas versus the urban areas probably due to the more heterogeneous composition in the sewage samples.

### 3.4. First-Derivative Synchronous Fluorescence Spectra

**Figure 4.**
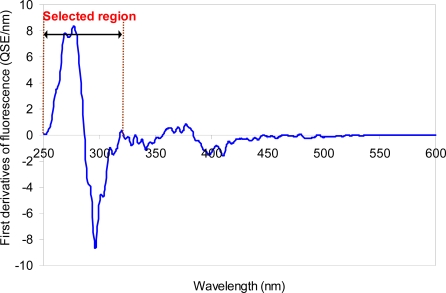
Typical first-derivative of synchronous fluorescence spectrum of the sewer sample from the sewer systems.

Similar to the zero-order spectra, there was no major difference in the first-order derivatives of the synchronous fluorescence spectra for the urban versus non-urban areas. The typical spectral pattern of the first derivatives is shown in Figure 4. The derivative spectra revealed two well-resolved peaks within a selected region at the wavelengths between 250 and 320 nm, which mostly corresponds to the PLF region. Regardless of the samples, one concave up and one concave down peaks were observed at the wavelength ranging from 250 to 290 nm and 290 to 330 nm, respectively.

### 3.5. Selection of Fluorescence Indices for BOD and COD Estimation

From the zero-order synchronous fluorescence spectra, the intensity of one peak in the PLF region (Index I) and another peak in the PLF region area (*i.e.*, fluorescence intensities multiplied by the selected wavelengths; Index II) were selected as the potential estimation indices for BOD. This is because PLF is reported to be associated with microbial activities and/or biodegradable organic matters [21]. The total area of the zero-order synchronous fluorescence spectra was also chosen as Index III for the BOD and COD prediction. From the first-derivatives of the spectra, the concave up peak (Index IV) and the whole area at the selected region were chosen as Index V. The correlation coefficients between the selected features and BOD or COD data were compared in Table 1.

All the selected fluorescence indices showed significant correlation coefficients with BOD and COD data (r > 0.776; p < 0.001). Irrespective of the selected indices, BOD estimation capability was consistently better for the urban areas compared to the non-urban areas as revealed by their higher correlation coefficients. This difference may be explained by the more homogeneous nature of sewage composition for the urban versus the non-urban areas. However, the same level of this difference was not observable for COD estimation. Index I presented the highest estimation capability for both BOD and COD based on all the sewage samples (Table 1).

**Figure 5.**
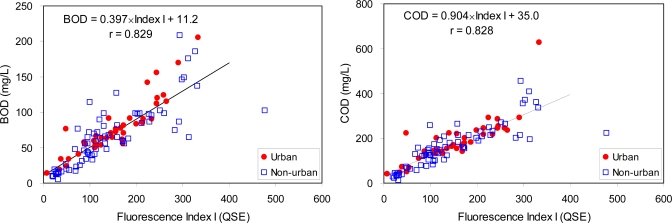
Correlations between a selected fluorescence index (Index I) and BOD or COD data.

Despite the high correlation coefficient, Index I was not effective to estimate BOD and COD for the high concentration ranges over 100 mg/L and 300 mg/L, respectively (Figure 5). Many data points were deviated from the regression lines between Index I and the bulk organic matter concentrations (BOD and COD) in the ranges. No distinction between the urban and the non-urban areas was found in regression lines. The final regression equations for BOD and COD were 0.397 × Index I + 11.2 and 0.904 × Index I + 35.0, respectively.

### 3.6. Optimization of BOD and COD Estimation using a Multi-Regression Method

**Figure 6.**
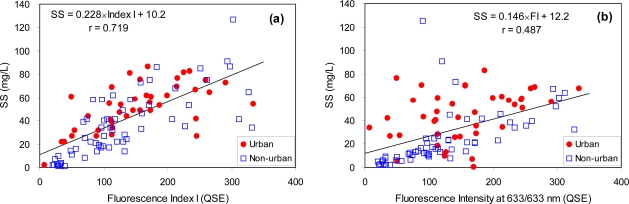
Correlations between Index I and suspended solid concentrations (SS) (a) and between Index I and the fluorescence intensity at excitation/emission wavelengths of 633/633nm (b).

One of the limitations in using fluorescence to estimate BOD and COD in sewage may be that the measured fluorescence is typically emitted from dissolved fluorescent organic matters, whereas a significant portion of BOD and COD concentrations originates from the suspended solids (SS) present in sewage samples. It is well-known that raw domestic wastewater contains about 45% to 55% of COD in the form of SS [22]. Although fluorescence intensities measured here were already amplified by the light scattering effect in measuring the unfiltered sewage samples, our selected fluorescence indices appear to be still insufficient to represent the SS contribution to BOD and COD. For example, the relationship between SS and Index I was much weaker than that obtained using BOD and Index I (Figure 6a). Although a recent study by Lee and Ahn [7] suggested that fluorescence intensity at the excitation/emission wavelengths of 633/633 nm may be replaced by SS concentrations in sewage samples, no such implication was found here, and in particular for the urban areas (Figure 6b).

**Figure 7.**
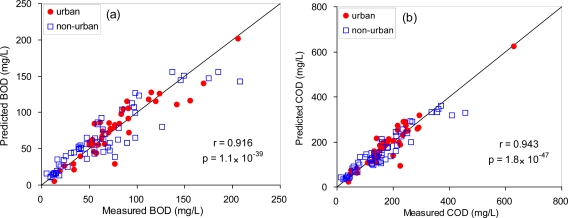
Correlation between measured BOD (left) and COD (right) and the predicted values by a multiple regression method using Index I and SS concentrations (n = 101, SS measurements were not taken for some samples).

For the present study, a multiple regression method based on Index I and SS concentrations was employed to compensate for the SS contribution and to improve the estimation capability for BOD and COD. As a result, the estimation capability was much enhanced with the correlation coefficients being 0.916 and 0.943 for BOD and COD, respectively (Figure 7). The regression equations using the multiple parameters were 0.455 × Index I + 0.089 × SS + 1.36 and 0.568 × Index I + 0.901 × SS + 14.26 for BOD and COD, respectively. The improvement in the estimation capability was more pronounced for COD. Moreover, the coefficient of SS in the regression equation was much higher for COD compared to BOD, indicating that the SS contribution to COD was greater than that of BOD. This also suggests that particulate matters in sewage may be more associated with non-biodegradable organic matters. To make the multiple regression approach available for real-time monitoring on-sites, however, it is necessary to present other methods to provide continuous SS data in sewage samples. More investigation needs to be undertaken to develop alternative real-time sensing techniques to fully reflect the contribution of SS in sewage to BOD and COD concentrations.

### 3.7. Potential Use of the Proposed Technique for In Situ Monitoring and the Limitations

Although the fluorescence data obtained here are based on a standard laboratory device, we expect that our proposed method will be utilized for the *in-situ* monitoring of wastewater quality in combined sewer systems in the future. It is because fluorescence sensing devices are easy to make in different sizes and at a desirable level of the signal-to-noise ratio according to the purpose of the work. Many handheld fluorometers with scanning function are now available, which allow one to use them as near-real time monitoring of wastewater quality of combined sewer systems. The limitation of this work may be that the data series investigated here are only based on wastewater samples collected during low-flow conditions. However, it should be noted that the matrices and the composition of the sewer vary extremely during rainfall runoff. In addition, the time series data during the high flow period should be much more valuable to evaluate the impacts on wastewater treatment plants and/or receiving water. In this sense, future work is required to prove the usefulness of the synchronous fluorescence spectrum for BOD and COD monitoring of storm water flows.

## 4. Conclusions

Different patterns in temporal changes of BOD and COD concentrations were found in domestic wastewater samples collected from the sewer pipes of urban and non-urban areas. However, the shapes of the synchronous fluorescence spectra were similar for the two land use types. Two distinct peaks were identified from the synchronous fluorescence spectra of the sewage samples. PLF peak is associated with aromatic amino acids and proteins, and the observed HLF peak appears to be related to the fluorescent whitening agents used in households. Among five selected fluorescence characteristics from synchronous fluorescence spectra and the first derivatives, the Index I—which correspond to a peak in the PLF region—exhibited the highest correlation coefficients (*r* = 0.83) with BOD and COD concentrations. The correlation with BOD was higher for the sewer samples from urban areas versus non-urban areas. A multiple regression approach based on SS and Index I revealed an improvement in the estimation capability for both BOD and COD. The correlation coefficients were improved from 0.83 to 0.92 and 0.94 for BOD and COD, respectively. The final estimation equations were 0.455 × Index I + 0.089 × SS + 1.36 and 0.568 × Index I + 0.901 × SS + 14.26 for BOD and COD, respectively.

## Figures and Tables

**Table 1.** The correlation coefficients between the selected fluorescence indices and the bulk organic matter parameters.

	**Types of area**	**Index I**	**Index II**	**Index III**	**Index IV**	**Index V**
BOD	Urban (n = 44)	0.900 (1.1 × 10^−15^)^a^	0.895 (3.0 × 10^−15^)	0.848 (1.3 × 10^−12^)	0.854 (1.3 × 10^−12^)	0.851 (1.7 × 10^−12^)
	Non-urban (n = 72)	0.804 (1.9 × 10^−17^)	0.789 (1.7 × 10^−16^)	0.782 (5.0 × 10^−16^)	0.763 (6.6 × 10^−15^)	0.803 (2.2 × 10^−17^)
	Total (n = 116)	0.829 (9.2 × 10^−30^)	0.813 (8.6 × 10^−28^)	0.783 (1.3 × 10^−24^)	0.774 (9.4 × 10^−24^)	0.815 (4.7 × 10^−28^)

COD	Urban (n = 44)	0.822 (4.6 × 10^−11^)	0.808 (1.7 × 10^−10^)	0.805 (2.2 × 10^−10^)	0.801 (3.1 × 10^−10^)	0.789 (9.2 × 10^−10^)
	Non-urban (n = 72)	0.839 (3.3 × 10^−20^)	0.826 (4.0 × 10^−19^)	0.839 (3.9 × 10^−15^)	0.790 (1.6 × 10^−16^)	0.838 (4.3 × 10^−20^)
	Total (n = 116)	0.828 (1.1 × 10^−29^)	0.812 (1.2 × 10^−27^)	0.808 (3.1 × 10^−27^)	0.776 (5.6 × 10^−24^)	0.818 (2.0 × 10^−28^)

a p value
